# Protocol for evaluating the nationwide implementation of the VA Stratification Tool for Opioid Risk Management (STORM)

**DOI:** 10.1186/s13012-019-0852-z

**Published:** 2019-01-18

**Authors:** Matthew Chinman, Walid F. Gellad, Sharon McCarthy, Adam J. Gordon, Shari Rogal, Maria K. Mor, Leslie R. M. Hausmann

**Affiliations:** 1 Veterans Integrated Service Network 4 Mental Illness Research, Education and Clinical Center, VA Pittsburgh, Pittsburgh, PA USA; 2 Center for Health Equity Research and Promotion, Veterans Affairs Pittsburgh, Pittsburgh, PA USA; 3 RAND Corporation, Pittsburgh, PA USA; 4 Department of Medicine, University of Pittsburgh, Pittsburgh, PA USA; 5 Program for Addiction Research, Clinical Care, Knowledge, and Advocacy, University of Utah School of Medicine, Salt Lake City, UT USA; 6 Informatics, Decision-Enhancement, and Analytic Sciences Center, VA Salt Lake City Health Care System, Salt Lake City, UT USA; 7 Department of Surgery, University of Pittsburgh School of Medicine, Pittsburgh, PA USA; 8 VA Pittsburgh Healthcare System, Research Office Building (151R), University Drive C, Pittsburgh, PA 15240 USA

**Keywords:** Implementation, Opioids, Facilitation

## Abstract

**Background:**

Mitigating the risks of adverse outcomes from opioids is critical. Thus, the Veterans Affairs (VA) Healthcare System developed the Stratification Tool for Opioid Risk Management (STORM), a dashboard to assist clinicians with opioid risk evaluation and mitigation. Updated daily, STORM calculates a “risk score” of adverse outcomes (e.g., suicide-related events, overdoses, overdose death) from variables in the VA medical record for all patients with an opioid prescription and displays this information along with documentation of recommended risk mitigation strategies and non-opioid pain treatments. In March 2018, the VA issued a policy notice requiring VA Medical Centers (VAMCs) to complete case reviews for patients whom STORM identifies as very high-risk (i.e., top 1% of STORM risk scores). Half of VAMCs were randomly assigned notices that also stated that additional support and oversight would be required for VAMCs that failed to meet an established percentage of case reviews. Using a stepped-wedge cluster randomized design, VAMCs will be further randomized to conduct case reviews for an expanded pool of patients (top 5% of STORM risk scores vs. 1%) starting either 9 or 15 months after the notice was released, creating four natural arms. VA commissioned an evaluation to understand the implementation strategies and factors associated with case review completion rates, whose protocol is described in this report.

**Methods:**

This mixed-method study will include an online survey of all VAMCs to identify implementation strategies and interviews at a subset of facilities to identify implementation barriers and facilitators. The survey is based on the Expert Recommendations for Implementing Change (ERIC) project, which engaged experts to create consensus on 73 implementation strategies. We will use regression models to compare the number and types of implementation strategies across arms and their association with case review completion rates. Using questions from the Consolidated Framework for Implementation Research, we will interview stakeholders at 40 VAMCs with the highest and lowest adherence to opioid therapy guidelines.

**Discussion:**

By identifying which implementation strategies, barriers, and facilitators influence case reviews to reduce opioid-related adverse outcomes, this unique implementation evaluation will enable the VA to improve the design of future opioid safety initiatives.

**Trial registration:**

This project is registered at the ISRCTN Registry with number ISRCTN16012111. The trial was first registered on 5/3/2017.

**Electronic supplementary material:**

The online version of this article (10.1186/s13012-019-0852-z) contains supplementary material, which is available to authorized users.

## Background

Drug overdoses have become the number one cause of accidental deaths in the United States (US). In particular, opioid overdose rates have surpassed the highest death rates from HIV, firearms, and motor vehicle accidents [1, 2]. Many have called the increase in opioid use and opioid-related adverse events a public health epidemic [3–7]. The increase in opioid medication prescribing has been a significant contributor to the increase in the incidence of drug overdose [8–12]. In 2014, 4.3 million people abused the prescription of opioids and 1.9 million people had an opioid use disorder (OUD) related to prescription opioids [13]. A seminal study in the Veterans Affairs (VA) Healthcare System demonstrated an association between receiving higher opioid doses and increased risk of opioid overdose death [9]. A similar risk has been demonstrated in non-VA settings [11, 12].

Opioid abuse is especially problematic among VA patients, as the annual prevalence rates are almost seven times higher than found in commercial health plans [14]. The number of Veterans receiving opioids at the VA almost doubled from 651,000 Veterans in 2001 to 1,101,346 in 2013. In 2013, 23% of VA pharmacy users received an opioid, up from 19% in 2001. Although opioid prescriptions have declined from 2013 to 2017 [15], safe prescribing remains a significant problem.

In 2013, VA launched the Opioid Safety Initiative to promote the safe and effective use of opioid analgesics. A major component of this initiative was the calculation and dissemination of monthly metrics on the average dose per day of select opioids and number of patients on concomitant opioids and benzodiazepines to facility and regional leaders (regions defined by 18 independent Veterans Integrated Service Networks (VISNs)) [16].

To augment these monthly metrics, the Office of Mental Health and Suicide Prevention (OMHSP) and the National Pain Management Program developed a predictive model to estimate the risk of serious adverse events (or SAEs, i.e., suicide-related events, overdoses, overdose deaths) among patients who are prescribed opioids. OMHSP translated the predictive model into the Stratification Tool for Opioid Risk Management (STORM). OMHSP developed STORM to empower facilities and clinicians to provide targeted opioid risk mitigation strategies to the most at-risk Veterans [17]. Using data from electronic medical records on demographics, previous overdose or suicide-related events, prescriptions, substance use and mental health disorders, and medical co-morbidities, the STORM algorithm calculates a “risk score” of SAEs for each Veteran who is prescribed opioids [17]. Updated nightly and based on the underlying predictive model, the STORM dashboard displays the estimated % chance of experiencing an SAE for each Veteran as well as their level risk categorized as low, medium, high or very high (for filtering purposes). The STORM dashboard also displays various risk factors that apply to each patient, including relevant diagnoses, prescriptions (opioid, pain, sedation), and use of risk mitigation strategies (including non-pharmacologic treatments). Finally, to support care coordination, the STORM dashboard also displays upcoming appointments to primary care, mental health, pain clinic, or other treatment sources.

In March of 2018, the VA released a national policy notice requiring all VA Medical Centers (VAMCs) to conduct data-based case reviews of patients with opioid prescriptions that STORM identifies as having very high risk of SAEs. Case reviews entail using the STORM dashboard and/or other data-based procedures (e.g., consulting state Prescription Drug Monitoring Programs) to evaluate each patient’s risk level and determine whether additional risk mitigation strategies (e.g., referral to pain specialist, prescription of naloxone kit) are indicated for that patient. The completed case review and any actions taken by the clinician are documented using a standardized note in the VA electronic medical record and in the STORM dashboard.

The dissemination of the policy notice and the impact of deploying STORM were selected as the focus of a randomized program evaluation for several reasons. First, the policy was motivated by the Commission on Care [18], a synthesis of an independent assessment of VA care, which recommended that VA, “Adopt a continuous improvement methodology to support VA transformation, and consolidate best practices...”. Also, the STORM dashboard is aligned with VA priorities to improve opioid safety, has the potential to fulfill the VA’s obligation to provide targeted opioid risk mitigation under the 2016 Comprehensive Addiction and Recovery Act (CARA; Public Law 114-198), and is ready for widespread implementation.

**Fig. 1:**
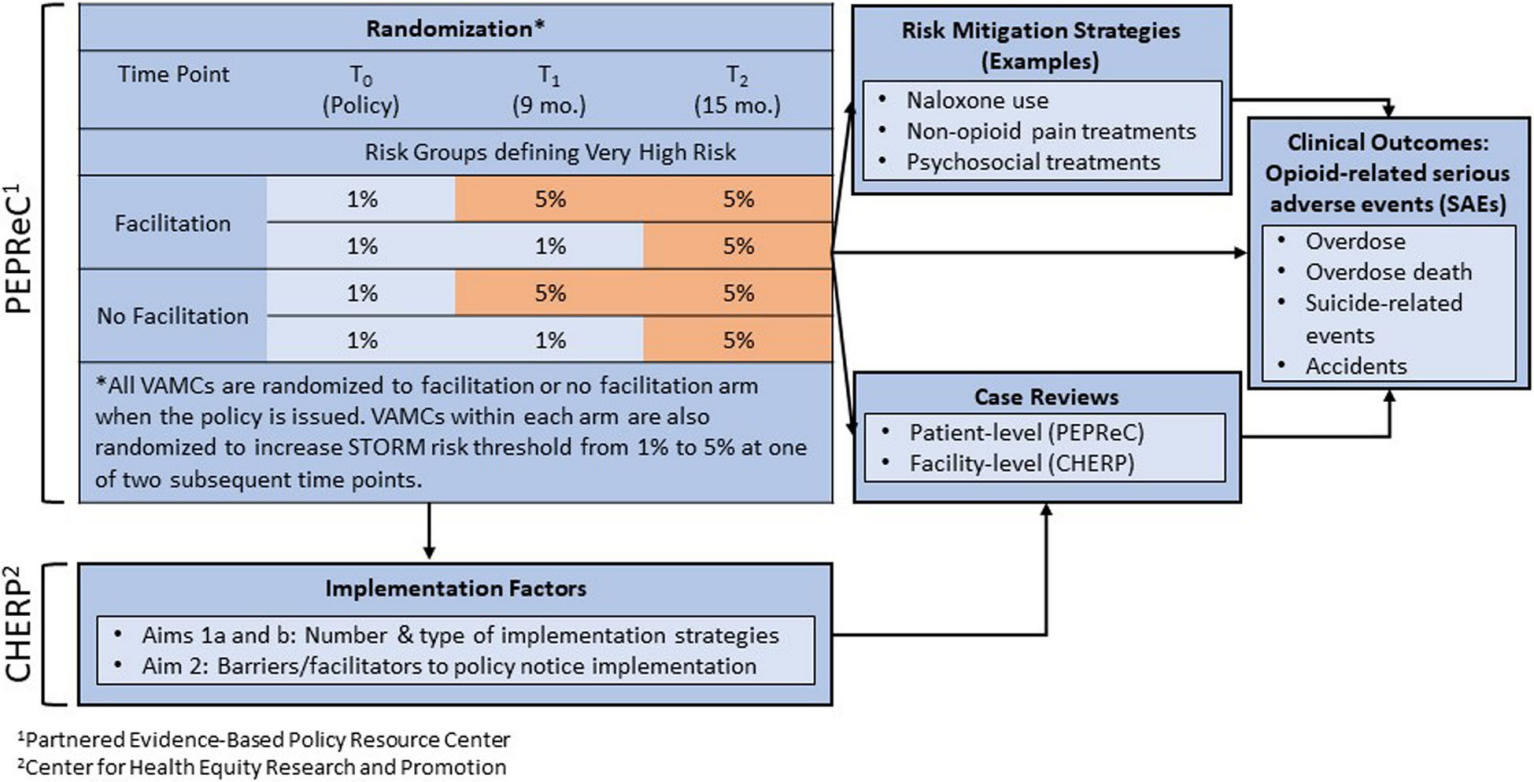
Design for clinical (PEPReC) and implementation evaluations (CHERP)

The STORM randomized program evaluation is being conducted by the VA Center for Health Equity Research and Promotion (CHERP), the Partnered Evidence-Based Policy Resource Center (PEPReC), and OMHSP. As described elsewhere and in more details below [19], the VA issued a policy notice requiring all VAMC’s complete data-based case reviews for patients whom STORM identifies as very high risk for SAEs (i.e., the top 1% of STORM risk scores). At random, half of the VAMC’s notices also stated there would be an additional support (called “facilitation” [20]) and oversight for VAMCs that failed to meet an established number of case reviews. Using a stepped-wedge cluster randomized design, VAMCs will be further randomized to conduct case reviews for an expanded pool of patients (top 5% of STORM risk scores vs. 1%) starting either 9 or 15 months after the notice was released, creating four natural arms. PEPReC will use VA administrative and clinical data to examine the effects of the facilitation and risk groups (1% vs 5%) on patient-level opioid-related SAEs (Fig. 1). PEPReC will also examine intermediate outcomes, including completion of STORM-facilitated case reviews and use of recommended opioid risk mitigation strategies and opioid and non-opioid pain therapies.

The focus of this report is the evaluation of implementation of the policy notice, led by an implementation evaluation team at CHERP. Specifically, the team will assess the number and type of implementation strategies VAMCs use to implement data-based case reviews of very high-risk patients as identified through STORM (aim 1a), and the association of implementation strategies with successful compliance with the STORM policy notice (aim 1b). The team will also examine barriers and facilitators to incorporating data-based case reviews into clinical practice (aim 2).

The opportunity to simultaneously track outcomes and to identify how implementation of the policy notice varies across VAMCs will advance VA’s ability to design and execute policies and programs in a way that improves care quality on a national level. In addition, the program evaluation has potential to advance implementation science by linking outcomes to specific implementation strategies.

## Methods

### Overview of STORM evaluation randomization design

All VAMCs have been randomized (March 2018) to STORM rollout arms that reflect two independent variables: (1) whether a VAMC receives additional oversight and assistance (i.e., facilitation: yes or no), and (2) the timing of when a VAMC’s group of patients to be reviewed will be expanded from the top 1% to the top 5% of STORM risk scores (i.e., risk group expansion: 9 or 15 months following the release of the policy notice) (Fig. 1). For the “facilitation” variable, VAMCs are randomized to one of two policy notices, both requiring the completion of data-based case reviews of patients identified by STORM as having a very high risk of SAEs. One policy notice specifies that, if fewer than 97% of the required case reviews have been conducted within 6 months of when the policy notice was released, the VAMC will be required to receive additional facilitation (i.e., outside coaching), which includes creating an action plan to implement case reviews and undergo additional oversight by OMHSP (the “facilitation” arm). The other policy notice includes no mention of additional oversight, action plans, or facilitation if the 97% case review metric is unmet (the “no facilitation” arm).

For the “risk group” variable, the pool of patients for whom case reviews are required will be expanded using a stepped wedge design. For a baseline period lasting 8 months following the release of the policy (*T*_0_), all VAMCs will be required to review all patients in the top 1% of STORM risk scores. At the beginning of month nine, the pool will be expanded at half of the VAMCs, at random, to include patients with STORM risk scores in the top 5% (*T*_1_). This step will last 6 months, at which time the pool of patients will be similarly expanded for the remaining VAMCs (*T*_2_). The change in risk group will occur “behind the scenes,” such that facilities are not aware which arm they are in or that the change is taking place. Varying whether the policy involves additional oversight and facilitation will enable an assessment of whether a policy that includes concrete repercussions for non-compliance is more effective than one that does not; varying the risk group defining which patients are required to have their cases reviewed will enable an assessment of the level of risk at which STORM-facilitated case reviews are associated with reduced risk of opioid-related SAEs. In both cases, there is equipoise about which arm will result in improved outcomes.

### Overview of the implementation evaluation

Per the policy notice, every VAMC will be required to identify a point of contact (POC) to oversee actions taken to carry out the policy. POCs could be pharmacists, physicians, or nurse managers and is expected to vary by site. POC information will be collected and maintained by OMHSP, who will share this information with the implementation evaluation team. The team will assess the number and type of strategies VAMCs use to implement case reviews following the randomized rollout of the policy notice and their association with case review completion rates (aims 1a and 1b). Specifically, we will use a survey based on a comprehensive set of implementation strategies, clustered into nine types of strategies (e.g., provide interactive assistance, train, and educate stakeholders), that were identified by implementation scientists as part of the Expert Recommendations for Implementing Change (ERIC) project [21, 22] (Additional file 1).

The implementation evaluation team will also collect information on the barriers and facilitators to incorporating the policy notice into clinical practice (aim 2). The Consolidated Framework for Implementation Research (CFIR) and other implementation models show that a wide range of factors at the individual (e.g., training, skills, efficacy, involvement in decision making) and organizational levels (e.g., size, climate, financial resources) influence implementation of evidence-based programs [23–27]. We will conduct CFIR-guided qualitative interviews with key stakeholders engaged in implementing the policy notice in a targeted sample of VAMCs to understand the variety of factors, both positive and negative, that affected implementation of the policy. As described below, the data from the quantitative ERIC survey and CFIR-based interviews will be considered as having been collected simultaneously [28] (as they are independent of each other) and then merged [29] to assess the match between barriers experienced and implementation strategies chosen across sites. All of the evaluation activities have been approved by the IRB at the Veterans Affairs Pittsburgh Healthcare System.

### Measures and data collection

#### Expert Recommendations for Implementing Change (ERIC) survey

##### Instrument

The survey used in the evaluation is based on the Expert Recommendations for Implementing Change (ERIC) project. Motivated by the need to develop a consistent set of terms for implementation strategies, ERIC engaged a panel of experts in implementation and clinical practice in a modified Delphi process to create consensus on 73 implementation strategies [21]. Concept mapping was then used to place the 73 strategies into nine clusters: Engage consumers, use evaluative and iterative strategies, change infrastructure, adapt and tailor to the context, develop stakeholder interrelationships, utilize financial strategies, support clinicians, provide interactive assistance, and train and education stakeholders. Each cluster contains 4 to 17 specific strategies [22]. Later, the ERIC strategies were used to create a survey administered to a POC from each VAMC for a national VA hepatitis C (HCV) initiative [30] aimed to promote adoption of new medication. The ERIC survey performed well in the HCV initiative [30]. Of those asked to complete the survey, responses were received from 133 (74%) individuals representing 80 VAMCs (62%). There was a moderate, positive correlation between the overall number of strategies and starts of the new medication (*r* = 0.43, *p* < 0.0001).

For the current evaluation, the ERIC survey’s items have been tailored to the policy notice and include 68 different implementation strategies representing all nine clusters; five items considered irrelevant to the policy notice were excluded. Respondents will be asked to indicate whether each strategy was used at their facility in the last 6–8 months (Yes or No), and if so, whether the strategy was implemented in direct response to the policy notice (Yes or No). Based on the survey’s use in the HCV initiative, we estimate that it will take approximately 30 min to complete the survey.

##### Data collection

Approximately 6 months following the release of the policy notice, the POC for the policy notice from every VAMC (*N* = 141) will be invited to complete the ERIC survey on behalf of their facility. The survey will be web-based and the web platform will ask respondents for consent before they complete the survey. To encourage high participation, OMHSP will first send an email to POCs alerting them that they will be contacted by the implementation evaluation team and asking that they consider participating in the evaluation. The importance of having all VAMCs represented in the analysis will be emphasized. The following week, the implementation evaluation team will send each POC an email that describes the ERIC survey, includes a facility-specific link to the online survey, and provides access to printable copy of the survey. POCs will be advised to review the survey in its entirety prior to completing the online version and to obtain input from others at their VAMC as needed. Study staff will follow up with POCs by email, followed by telephone calls, and/or the VA’s instant messaging system, as needed. Reminders to complete the survey will also be made at the monthly Community of Practice calls hosted by OHMPSP during the recruitment period.

##### Facility-level covariates

Depending on data availability, we plan on examining several facility characteristics for descriptive purposes and to explore their association with the use of case review implementation strategies and completion rates. Following Glasgow et al.’s analytic framework of organizational factors, [31] indicators of two broad dimensions of organizational context will be examined: facility structure (e.g., number of annual primary care visits, patient clinical severity, facility complexity, region) and staffing/culture (e.g., primary care patient panel size, primary care staff ratio, number of mental health staff, specialty pain program workload, use of academic detailing for opioid safety, and several scales from the VA All Employee Survey) (Table 1). Facility structure variables refer to basic, unmodifiable structural characteristics, whereas staffing/culture variables include more modifiable characteristics that may affect the success of a facility’s QI efforts. All facility structure characteristics will be extracted from VA administrative data sources.

**Table 1:** Definitions of facility characteristics planned for inclusion

Facility characteristic	Definition (data source)	Variable type
Facility structure		
Number of annual primary care visits	Total number of outpatient visits in primary care clinics (CDW)	Quartiles
Patient severity	VA calculates patient severity using a Nosos Risk Score, which is based on a risk adjustment model developed by Center for Medicaid & Medicare Services and further calibrated with VA pharmacy data, VA priority status, and VA-computed costs [41]. Scores are scaled such that the mean Nosos score = 1, so scores > 1 indicate greater-than-average cost and clinical complexity. (VA PACT Compass*)	Continuous, scaled by 0.10
Facility complexity	Based on an algorithm that takes into account patient risk, number and breadth of available specialists, intensive care unit availability, and teaching and research activities. (VA Office of Productivity, Efficiency, and Staffing)	1a, 1b, 1c, 2, or 3 (1a = most complex)
Region	Region of country defined by Census (HHS Area Resource File)	Northeast, Midwest, South, or West
Staffing/Culture		
Primary care patient panel size	Average panel size of primary care providers (VA PACT Compass*)	Continuous, scaled by 100
Primary care staff ratio	Number of primary care support staff divided by the number of direct-care primary care providers (medical doctors or other direct-care providers such as nurse practitioners). Higher staff ratios indicate more support staff per direct-care provider, with 3 being the recommended minimum ratio for successful PACT implementation. (VA PACT Compass*)	Continuous
Mental health staff	Number of mental health full time equivalents relative to enrollees (Office of Mental Health and Suicide Prevention)	Continuous
Specialty pain program workload	Number of unique patients with at least one pain clinic visit (stop code 420) relative to enrollees (Office of Mental Health and Suicide Prevention)	Continuous
Academic detailing	Number of staff who undergo academic detailing on STORM, specifically, and other topics related to opioid risk management (SalesForce Database)	Continuous or categorical, as appropriate
Workgroup psychological safety	Degree to which employees agree that members of their workgroup are able to bring up problems and tough issues (1 = strongly disagree; 5 = strongly agree). Measured using a single item from the 2018 All Employee Survey.	Continuous
Servant leadership	Summary measure calculated from 5 items from the 2018 All Employee Survey assessing whether the work environment is a place where organizational goals are achieved by empowering others. Scores range from 0 to 100, with higher scores being more favorable.	Continuous
Raise and discuss ethics	Degree to which employees agree that their direct supervisor raises and discusses ethical concerns (i.e., uncertainty or conflict about the right thing to do) (1 = strongly disagree; 5 = strongly agree). Measured using a single item from the 2018 All Employee Survey.	Continuous
Workplace performance	“Workplace Performance” is a summary measure calculated using 6 items from the 2018 All Employee Survey that assess the degree to which the workplace environment has the right resources, training, goals, and innovation in place to support optimal performance. Scores range from 1 to 5, with higher scores being more favorable.	Continuous

*CDW* Corporate Data Warehouse, *DCG* Diagnostic Cost Group, *FY* fiscal year, *IOC* independent outpatient clinic, *VSSC* VA Support Service Center

*VA PACT Compass is an electronic dashboard of metrics that reflect the extent to which facilities are successful in providing team-based, continuous, and coordinated care

#### Consolidated Framework for Implementation Research (CFIR)-based interviews

##### CFIR interview protocol

The CFIR is a predominant framework in implementation science [32], containing a comprehensive listing of factors that have been shown to influence implementation of new programs and initiatives. Working from the qualitative questions developed by CFIR developers (https://cfirguide.org/), we created an interview protocol that focuses on four CFIR domains: (1) intervention characteristics (e.g., strength, quality), (2) outer settings (e.g., external policies, incentives), (3) inner settings (e.g., clinic culture), and (4) the implementation process (e.g., engagement of clinic providers). See Table 2 for the domains and subdomains of the CFIR included in the protocol. No data will be collected for the fifth CFIR domain, characteristics of individuals, as the focus of this research is on collective barriers and facilitators, not individual-level factors. The interview protocol adapted questions to assess key elements of how the case review process was implemented at a site. The implementation evaluation team reviewed the interview protocol and iteratively suggested changes and improvements. The protocol will be pilot tested in two sites determined to be outside the sites targeted for final interviews.

**Table 2:** Consolidated Framework for Implementation Research (CFIR) interview domains, subdomains, and example questions

Domain: intervention characteristics	
*Evidence strength and quality:* Can you tell me about any evidence that you are aware of that shows that completing case reviews for high risk patients will result in better outcomes for Veterans?	
*Relative advantage*: How does the case review process required by the policy notice compare to other similar existing processes or tools in your setting?	
Domain: outer setting	
*Patient needs and resources*: How well do you think the policy notice is helping to meet the needs of Veterans?	
*Peer pressure*: Are you aware of how your team is doing compared to other medical centers and CBOCS in complying with the policy notice?	
*External policies and incentives*: Why do you think the policy notice was implemented?	
Domain: inner setting	
*Structural characteristics*: Tell me about any changes that were needed to accommodate the policy notice?	
*Networks and communications*: Can you describe your relationship with influential stakeholders and others who have worked with you to implement the policy notice?	
Implementation Climate	
*Tension for change*: Why do you think the policy notice was implemented?	
*Compatibility*: Describe how doing case reviews for high risk Veterans has been integrated into the current workflow.	
*Relative priority*: Compared to other high-priority initiatives going on at your medical center, how important was it to comply with the policy notice?	
*Goals and feedback*: What goals did your facility have in place to reduce adverse outcomes from opioid use prior to the implementation of the policy notice?	
*Leadership engagement*: What level of endorsement or support have you seen or heard from your local leadership?	
*Available resources*: What resources have you needed to implement and carry out the policy notice?	
*Access to knowledge and information*: What kind of training did you receive about the policy notice?	
Domain: process	
*Planning*: Were you involved in developing a plan for implementing the policy notice at your facility?	
*Engaging*: What steps were taken to encourage individuals to perform case reviews for Veterans identified as high risk by STORM?	
*Opinion leaders*: What key individuals needed to be on board with the policy notice?	
*Formally appointed internal implementation leaders*: Tell me about your role in the implementation of the policy notice.	
*Reflecting and evaluating*: Tell me about the feedback reports that you receive from VA central office about your progress implementing the policy notice.	

##### Data collection

We will conduct the CFIR-based interviews with key informants from a purposeful sample of 40 facilities to understand the barriers and facilitators to implementation of the policy notice. These interviews will be conducted at the five VAMCs in each evaluation arm with the highest and lowest adherence to opioid therapy guidelines as measured by the Opioid Therapy Guideline (OTG) Adherence Report in the year prior to the release of the policy notice [33]. The OTG report contains a set of national metrics that examine facility-level performance on guideline-recommended opioid practices. Our team will recruit the policy notice POC at each facility (the same person contacted to complete the ERIC survey, or their replacement if the original POC is no longer in place) via email and/or telephone. If the current POC or an appropriate replacement is not available at a given VAMC, we will identify additional facilities in the order of their OTG adherence scores. Each facility POC will be asked to complete the interview and identify one or two additional individuals to be interviewed, based on who completed the most case reviews at that facility. The key informant plus the one or two individuals they recommend will form the interview sample at each facility, for a maximum of 120 telephone interviews. The 45-min interviews will be conducted via telephone by trained interviewers from the implementation evaluation team. Interviews will be audio-recorded and transcribed verbatim. While we considered having interviewees review their transcripts, research has shown that the errors that tend to get corrected are often minor, yet the process invites other methodological problems (e.g., interviewees changing their answers after the fact) [34]. Transcripts Interviewers will be blinded to randomization arm. The interviews will occur approximately 10–12 months after the policy notice release, which will provide separation from the ERIC survey in aim 1 (to reduce respondent burden) and allow us to identify how barriers and facilitators may differ after the risk thresholds are changed across randomization arms (*T*_1_ in Fig. 1).

### Data analysis

#### Aim 1

ERIC survey responses will first be summarized by computing a cross tabulation of use of each of the 68 strategies in the past 6–8 months (yes/no) by the two facilitation randomization arms (facilitation or no facilitation). The proportion of strategies used within each cluster of strategy types and the total number of strategies implemented in the past 6–8 months will also be computed and described by arm. This process will be repeated to describe the number and type of strategies that were implemented as a direct response to the policy notice. Primary analyses for aims 1a and 1b will be based on whether the strategy was used in the past 6–8 months. As a sensitivity analyses, we will repeat these analyses based on whether the strategy was implemented as a direct response of the policy notice, which will be indicated in the survey responses.

#### Aim 1a

We will test for significant differences between the number of strategies implemented overall and by facilitation arm using the Kruskal-Wallis test. This will test if there are systematic differences in the use of more strategies in one arm, compared to the other, and does not assume that the total number of strategies implemented is normally distributed. Differences in the predominant cluster of strategy type implemented between the two arms will be computed using exact chi-square tests for the cross tabulation of type of strategy by arm, described above.

#### Aim 1b

The primary analysis for aim 1b will estimate the association between number of implementation strategies used and completion of case reviews at *T*_0_ + 6 months. If enough VAMCs meet the 97% target completion rate, we will analyze the data using logistic regression for the outcome of meeting the target rate; otherwise, we will model the proportion of case reviews completed. The outcome will be modeled using a generalized linear model with logit link and binomial family. This strategy will appropriately model the case review completion rates as being restricted between 0% and 100% and is equivalent to logistic regression for binary (yes/no) outcomes. For these analyses, the number of implementation strategies used will be the independent variable and will be coded as either continuous or categorical. Because difficulty in completing case reviews could increase as volume of very high-risk patients increases, we will include the number of very high-risk patients at each facility as a predictor along with the other facility variables.

Additional analyses will assess the association between the type of implementation strategies used and the case completion rate. We will assess the association between each individual strategy and case review completion in individual regression analyses. We will also assess the relationship between the percent of implementation strategies used within each cluster of strategy types and case review completion rates. Because we have adapted the ERIC survey for this evaluation, we will conduct confirmatory factor analyses to verify that the original clusters were maintained. If the factor analysis does not confirm the original clusters, we will describe the resulting strategy type factors and utilize summary scores based on the new factors. Finally, we will assess whether the overall number of implementation strategies used mediates the relationship between randomization arm and case review completion rate. We will test mediation using the bootstrapping methods of Preacher and Hayes [35]. These methods will allow for the direct estimation and testing of mediation and do not make any distributional assumptions (such as normality) regarding the variables or the test statistic. Analyses are based on taking a large number of bootstrap samples with replacement from the original data and estimating effects for each sample. The mean estimated effect and empiric confidence intervals are computed across all samples. Furthermore, because inferences do not depend upon large-sample theory, this method can be used for data with smaller sample sizes.

##### Power and sample size

We computed power and sample size calculations using Pass 14 (Kaysville, UT). Based on the ERIC survey data from the HCV initiative [30], we anticipate receiving responses from at least 80 facilities and assume that the mean number of strategies implemented in the no facilitation arm will be 25 strategies with a standard deviation of 14. For aim 1a, we will have 80% power to detect a difference of 9.15 ERIC implementation strategies between the study arms using an alpha level of 0.05. Calculations are similar when allowing for different sample sizes with the ability to detect differences of 8.6 (90 facilities) to 9.75 (70 facilities) for varying facility responses. For aim 1b, the detectable difference in case review completion rates depends upon the sample size and the baseline completion rate in the no facilitation arm. Table 3 provides the difference in case review completion rates detectable in the facilitation arm for a variety of baseline completion rates and sample sizes. For example, assuming a 70% baseline completion rate and a sample size of 90, we have 80% power to detect an 11.6 percentage point difference in the case review completion rate in the facilitation arm, which is equivalent to an odds ratio of 1.91.

**Table 3:** Power calculations for aim 1b

Baseline rate (%)	Sample size					
	70		80		90	
	∆* (%)	OR	∆* (%)	OR	∆* (%)	OR
50	+ 16.1	1.95	+ 15.2	1.87	+ 14.4	1.81
60	+ 14.8	1.98	+ 14.0	1.90	+ 13.3	1.83
70	+ 12.9	2.08	+ 12.2	1.98	+ 11.6	1.91
80	+ 10.2	2.31	+ 9.7	2.19	+ 9.3	2.09
90	+ 6.5	3.05	+ 6.2	2.84	+ 6.0	2.68

*Detectable difference from baseline case completion rate in the consequences (vs. no consequences) arm at a given sample size and with 80% power

#### Aim 2

We will use CFIR-based interviews to identify barriers and facilitators to implementing the policy notice and compare these barriers and facilitators across facilities with high and low adherence to recommended opioid therapy guidelines in each of the four randomization arms. Codebook construction will follow methods outlined by Crabtree and Miller for research conducted in a medical context [36]. We will employ a system of audit trails to document the creation of codes. For each code, the codebook will specify inclusion/exclusion criteria and textual examples of clear and borderline cases. Representative quotations will be captured verbatim from the transcripts using Atlas.ti. As part of the coding process, the analysts will meet and process any differences in the assessment of codes for each case until agreement is achieved. The codes determined through this agreement process will then be recorded in a master file, which will become the basis for the final analysis. This process of coding independently (the basis for the inter-coder reliability scores) and then discussing each case will aid in achieving high levels of inter-coder reliability.

In addition to qualitative data analysis, the coded text data from the CFIR interviews will be used to make the numerical ratings for the individual subdomains under the four domains included. In general, ratings are determined based on two aspects: (1) valence (positive or negative impact on implementation) and (2) strength (the degree to which implementation was facilitated or hindered, with possible choices being 0, 1, or 2) [20]. The valence component of a rating is determined by the influence the coded data exhibits on the implementation process; that is, is the subdomain in question broadly facilitating or hindering? The strength of a rating has several determinants, including level of agreement among participants, strength of language, use of concrete examples, and stated influence on implementation or another subdomain. Considering both the valence and the strength of each subdomain, the rating scale ranges from + 2 (most facilitating) to − 2 (most hindering). A zero rating reflects a neutral (e.g., statements lacking sufficient information to make a clear scoring distinction) or mixed influence of the code (e.g., statements indicating equal presence and absence of a subdomain and having no impact on implementation). A team will meet and review all the comments associated with a specific construct, and then assign a rating. Each construct will be rated for each arm of the study, allowing for detailed descriptions of barriers and facilitators across arms. This CFIR rating process also creates an overall picture of how the domains differ across arms, providing guidance for future implementation efforts.

#### Merging data from aims 1 and 2

In addition to separately analyzing the ERIC survey data and the CFIR-based interviews, we will merge these two data sources as described by Creswell & Plano Clark [29]. This integration will be done to assess the degree to which the most common implementation strategies that significantly predict case review completion rates are matched with certain barriers identified by the CFIR interviews. Then, these results could be compared to the work of Damschroder, Powell, and colleagues [37] who used an expert panel to begin to determine which strategies are best suited to address barriers across various CFIR domains.

## Discussion

Examining the implementation process of clinical practices has become an essential part of evaluating the impact of such a practice [24]. Often healthcare policies make recommendations about what to do without including specific guidance about how to address system-level implementation barriers. Along these lines, the STORM policy notice has allowed VAMCs and individual providers to determine whether or how to go about conducting case reviews and whether or not to use the STORM dashboard. Although OMHSP has offered general guidance, webinars about STORM’s availability, and guidance through the policy notice itself on how to carry out the case reviews (e.g., identifying a POC, suggesting staff to be involved, establishing a specific case review note), providing in-depth facilitation to all VAMCs is not practical. Also, there are several models that VAMCs could use to incorporate data-based case reviews into practice, and it is likely that VAMCs will vary in the models they use based on their local environment. Complicating matters further, VA, the largest integrated healthcare system in the US, includes facilities that vary widely on myriad factors such as size, location, patient populations, resources, services offered, and organization, all of which could affect implementation of the policy notice. Local variability has been seen in other related national initiatives, such as implementation of buprenorphine for the treatment of opioid use disorder [38–40].

OMHSP is interested in understanding whether and how the case reviews of patients at very high risk of opioid-related SAEs get incorporated into practice and the factors that affect the ability of VAMCs to carry out such case reviews effectively. Understanding whether including facilitation (vs. no facilitation) in the policy notice increases facility compliance will inform future policy development. Testing the effect of adding new patients into the STORM risk group for whom case reviews are required will yield insights regarding which patients should be targeted for case reviews to achieve the desired reduction in opioid-related SAEs. The implementation evaluation will complement the analysis of changes in opioid-related SAEs over time and across randomization arms. We will be able to learn which implementation strategies are most effective in supporting case reviews, and how that relationship differs between the facilitation (which itself is an implementation strategy) and no facilitation arms. We will also be able to learn which barriers and facilitators, using CFIR, are most salient across the four arms. Finally, combining data on implementation strategies and barriers at such a large scale could advance implementation science by showing which strategies are naturally best suited to address certain barriers.

There are important limitations that also must be considered. Most importantly, this intervention is occurring in the context of many other national, regional, and local opioid safety initiatives emerging or already in place. The randomized design is an important method to address this concern, and the ERIC survey and qualitative interviews will elucidate how other initiatives impact particular facilities during the course of the trial. Second, any survey targeting employees and providers will have challenges achieving an adequate response rate, especially given the length of the survey and inability to incentivize respondents. However, the previous use of the ERIC survey in a similar initiative achieved a good response rate (74%) and found that respondents who started the survey completed the survey, with no bias towards endorsement of strategies presented earlier in the survey [30]. Finally, the evaluation is at the facility level, meaning there will be ERIC data on no more than 141 sites. Power is thus limited to detect small differences across arms and to control for all the potentially relevant organizational covariates. However, we are maximizing the chance of finding an effect by using a rigorous randomized approach that includes all VAMCs.

In sum, the evaluation of implementation factors will provide valuable insights into how VAMCs implement a policy designed to reduce opioid-related SAEs among Veterans and will identify types of implementation strategies that are associated with successful implementation. The resulting data from the evaluation described here will help OMHSP and other VA decision makers prioritize which types of implementation strategies to recommend and use in future policy rollouts and implementation initiatives.

### Trial status

This evaluation was funded as a VA Health Services Research and Development Service Directed Project in April of 2016 and has proceeded in two phases. In the planning phase (April 2016–March 2017), the implementation evaluation team met biweekly with representatives from OMHSP and PEPReC to inform the language of the policy notice, to determine the design and timing of the arms to be included in the stepped-wedge randomized evaluation, and to align the activities that would be completed by each partner (OMHSP, PEPReC, and our implementation evaluation team) leading up to and following the release of the policy notice. These biweekly discussions have continued since transitioning to the active evaluation phase (April 2017–March 2020) and have maintained awareness and alignment of activities across each partner.

The implementation evaluation activities were approved by the Veterans Affairs Pittsburgh Healthcare System Institutional Review Board on March 6, 2017. The policy notice VA NOTICE 2018-08 CONDUCT OF DATA-BASED CASE REVIEWS OF PATIENTS WITH OPIOID-RELATED RISK FACTORS, was released to the field in March of 2018, at which time VAMCs (*N* = 141) were randomized to one of four evaluation arms. Collection of the ERIC survey data for aim 1 began October 1, 2018 and closed December 16, 2018, at which time OMHSP began reaching out to VAMCs that have not reached the 97% case review completion rate target. If survey responses are received after that date, they will be flagged as being received after facilitation began at some sites and will be explored to determine whether they differ from responses received earlier. Recruitment of VAMCs for CFIR interviews (aim 2) will begin in February of 2019.

## Additional file

Additional file 1: Survey of VHA Notice 2018-08 Implementation Strategies. (DOCX 49 kb)

## Abbreviations

CFIR: Consolidated Framework for Implementation Research
CHERP: Center for Health Equity Research and Promotion
ERIC: Expert Recommendations for Implementing Change
OMHSP: Office of Mental Health and Suicide Prevention
OTG: Opioid Therapy Guidelines
PEPReC: Partnered Evidence-Based Policy Resource Center
POC: Point of contact
STORM: Stratification Tool for Opioid Risk Management
VAMC: Veterans Affairs Medical Centers
