# Supplementary material for: Protocol for evaluating the nationwide implementation of the VA Stratification Tool for Opioid Risk Management (STORM)
Source: Implement Sci. 2019 Jan 18;14:5. doi: 10.1186/s13012-019-0852-z (PMC6339438; doi:10.1186/s13012-019-0852-z)
Supplement: Supplementary file 1 — Survey of VHA Notice 2018-08 Implementation Strategies. (DOCX 49 kb) [file 13012_2019_852_MOESM1_ESM.docx]

**Survey of VHA Notice 2018-08 Implementation Strategies**

Thank you for participating!

This survey will ask about implementation strategies that your VA medical center may be using to achieve the goal set forth by *VHA Notice 2018-08: Conduct of Data-Based Case Reviews of Patients with Opioid-Related Risk Factors* — namely, completing case reviews of patients at very high risk for opioid-related adverse events. You have been chosen to take this survey on behalf of your medical center because you are the point of contact on record with the Office of Mental Health and Suicide Prevention for overseeing your medical center’s response to *VHA Notice 2018-08.*

An implementation strategy is any action that could be taken to increase the successful utilization of a certain practice (in this case, completion of case reviews mandated by *VHA Notice 2018-08*). Because there are many types of implementation strategies, it is not likely that any one medical center would use all of them. The goal of this survey is to understand which implementation strategies are, and are not, being used at your medical center.

On the following pages, you will be presented with a series of specific implementation strategies.

- First, you will be asked to respond YES or NO as to whether a strategy was used in the last 6-8 months.
- If you respond YES, you will then be asked whether your medical center implemented the strategy or increased its use in direct response to *VHA Notice 2018-08*. If you respond NO, you will move on to the next strategy.

Your survey is the only one being collected for your medical center, so it is important that you do your best to provide a complete and accurate picture of your medical center’s activities. We do not expect that all strategies will be used, so do not feel as though you need to answer YES to every question. **We encourage you to reach out to other individuals at your medical center if you need more information to answer any of the questions.**

You can pause the survey and continue where you left off as many times as needed by using the “Save & Return Later” button at the bottom of the screen. Be sure to copy or write down the Return Code given after clicking on the button. Without it, you will not be able to return and continue the survey where you left off.

If you need further assistance, you may also reach out to Dr. Matthew Chinman (Matthew.Chinman@va.gov; 412-683-2300 x4287) or Dr. Leslie Hausmann (Leslie.Hausmann@va.gov; 412-360-2112) from the STORM Implementation Program Evaluation Team.

Click “Next Page” when you are ready to begin.

**Evaluative and Iterative Implementation Strategies**

| **In the last 6-8 months, did your VA medical center use any of these evaluative and iterative strategies to promote completion of mandated case reviews of patients at very high risk for opioid-related adverse events?** | **Was this strategy used in the last 6-8 months?** | **[If yes] Was the above strategy implemented in direct response to *VHA Notice 2018-08*?** |
| --- | --- | --- |
| 1. Assess readiness to change | No Yes | No Yes |
| 1. Collect data on the completion of mandated case reviews of very high risk patients and use it to provide feedback and modify behavior | No Yes | No Yes |
| 1. Implement small changes in ways to improve completing mandated case reviews of very high risk patients, then assess those changes using performance data before making system-wide changes | No Yes | No Yes |
| 1. Collect and analyze information about the resources that your facility would need to complete mandated case reviews of very high risk patients | No Yes | No Yes |
| 1. Develop a written implementation plan including goals and strategies | No Yes | No Yes |
| 1. Develop and use local quality-monitoring systems that could include standards, protocols, or other tools | No Yes | No Yes |
| 1. Obtain and use patient or family feedback on having their case reviewed for opioid risk management | No Yes | No Yes |
| 1. Regular monitoring and adjusting practices (as needed) for completing mandated case reviews of very high risk patients | No Yes | No Yes |
| 1. Start with small pilot initiatives to increase the rate of completing mandated case reviews of very high risk patients and then scale them up | No Yes | No Yes |

**Provide Interactive Assistance**

| **In the last 6-8 months, did your VA medical center employ any of these activities to provide interactive assistance to promote completion of mandated case reviews of patients at very high risk for opioid-related adverse events?** | **Was this strategy used in the last 6-8 months?** | **[If yes] Was the above strategy implemented in direct response to *VHA Notice 2018-08*?** |
| --- | --- | --- |
| 1. Assign someone from the local medical center to help relevant healthcare professionals with the clinical issues involved in completing mandated case reviews of very high risk patients (sometimes called "technical assistance") | No Yes | No Yes |
| 1. Use a centralized system, for example from the VISN, to deliver technical assistance | No Yes | No Yes |
| 1. Use an outside facilitator to provide coaching, education, or other help with implementation | No Yes | No Yes |
| 1. Provide supervision on how to complete mandated case reviews of very high risk patients | No Yes | No Yes |

**Adapt and Tailor to the Context**

| **In the last 6-8 months, did your VA medical center employ any of these activities to tailor how mandated case reviews of patients at very high risk for opioid-related adverse events are completed?** | **Was this strategy used in the last 6-8 months?** | **[If yes] Was the above strategy implemented in direct response to *VHA Notice 2018-08?*** |
| --- | --- | --- |
| 1. Identify ways that the process of completing case reviews of very high risk patients can be adapted to meet local needs while still maintaining the core components of the review process | No Yes | No Yes |
| 1. Tailor strategies to complete case reviews of very high risk patients to address specific barriers identified in your medical center | No Yes | No Yes |
| 1. Use the STORM dashboard to facilitate completion of mandated case reviews | No Yes | No Yes |
| 1. Use data from sources other than the STORM dashboard, for example OTTR or the Corporate Data Warehouse, to facilitate completion of mandated case reviews | No Yes | No Yes |
| 1. Consult data experts to help your medical center use or understand data about your patients who are prescribed opioids | No Yes | No Yes |

**Develop Stakeholder Interrelationships**

| **In the last 6-8 months, did your VA medical center employ any of these activities to develop stakeholder interrelationships to promote completion of mandated case reviews of patients at very high risk for opioid-related adverse events?** | **Was this strategy used in the last 6-8 months?** | **[If yes] Was the above strategy implemented in direct response to *VHA Notice 2018-08*?** |
| --- | --- | --- |
| 1. Recruit and cultivate relationships with local partners in your medical center to facilitate the completion of mandated case reviews | No Yes | No Yes |
| 1. Document and share with other medical centers knowledge gained from local efforts to complete mandated case reviews | No Yes | No Yes |
| 1. Include local relevant healthcare professionals and other stakeholders in discussions of whether conducting mandated case reviews is an appropriate method of opioid risk management | No Yes | No Yes |
| 1. Partner with a university to share ideas about completing case reviews of very high risk patients | No Yes | No Yes |
| 1. Develop and distribute a glossary of terms that relevant healthcare professionals might not be familiar with but are important for completing mandated case reviews | No Yes | No Yes |
| 1. Select or train local relevant healthcare professionals who will dedicate themselves to promoting the completion of mandated case reviews of very high risk patients | No Yes | No Yes |
| 1. Identify those who were quick to start case reviews once asked (early adopters) to learn from their experiences completing mandated case reviews | No Yes | No Yes |
| 1. Inform local opinion leaders about the need to complete case reviews of very high risk patients (e.g., clinical/administrative leaders or other influential colleagues) | No Yes | No Yes |
| 1. Involve the medical executive board or other existing governing structures in supporting progress towards the completion of mandated case reviews | No Yes | No Yes |
| 1. Simulate the completion of mandated case reviews to identify potential problems | No Yes | No Yes |
| 1. Obtain formal written commitments from key local stakeholders that state what they will do to support the completion of mandated case reviews, for example written agreements with CBOCs or between service lines | No Yes | No Yes |

**Develop Stakeholder Interrelationships (continued)**

| **In the last 6-8 months, did your VA medical center employ any of these activities to develop stakeholder interrelationships to promote completion of mandated case reviews of patients at very high risk for opioid-related adverse events?** | **Was this strategy used in the last 6-8 months?** | **[If yes] Was the above strategy implemented in direct response to *VHA Notice 2018-08*?** |
| --- | --- | --- |
| 1. Provide protected time to allow people to complete mandated case reviews to meet and share lessons | No Yes | No Yes |
| 1. Capitalize on existing high-quality networks to promote information sharing and problem solving related to implementing mandated case reviews | No Yes | No Yes |
| 1. Recruit, designate, or train leaders specifically to manage the completion of mandated case reviews at their medical center | No Yes | No Yes |
| 1. Seek input from advisory boards and interdisciplinary workgroups on ways to complete mandated case reviews of very high risk patients | No Yes | No Yes |
| 1. Seek the guidance of experts on implementing change in healthcare settings | No Yes | No Yes |
| 1. Visit sites outside your medical center that have been successful at completing mandated case reviews to learn from their experiences | No Yes | No Yes |

**Train and Educate Stakeholders**

| **In the last 6-8 months, did your VA medical center employ any of these activities to train or educate stakeholders to promote completion of mandated case reviews of patients at very high risk for opioid-related adverse events?**  **All items below can refer to education or training about *clinical content* (e.g., what clinical factors put a patient at high risk for an opioid overdose), the *case-review process* (e.g., how to search the STORM dashboard), or both.** | **Was this strategy used in the last 6-8 months?** | **[If yes] Was the above strategy implemented in direct response to *VHA Notice 2018-08*?** |
| --- | --- | --- |
| 1. Conduct an initial training session | No Yes | No Yes |
| 1. Provide ongoing training | No Yes | No Yes |
| 1. Have an outside expert in completing the mandated case reviews meet with relevant healthcare professionals to provide general trainings one-on-one or in groups | No Yes | No Yes |
| 1. Have an outside expert meet with relevant healthcare professionals to provide case-specific guidance and feedback | No Yes | No Yes |
| 1. Create or participate in groups that meet regularly to discuss and share lessons learned | No Yes | No Yes |
| 1. Develop local educational materials, like guidelines, manuals, or toolkits | No Yes | No Yes |
| 1. Distribute relevant educational materials | No Yes | No Yes |
| 1. Vary education or training methods to cater to different learning styles | No Yes | No Yes |
| 1. Give relevant healthcare professionals opportunities to shadow or otherwise observe experts conducting mandated case reviews of very high risk patients | No Yes | No Yes |
| 1. Train designated relevant healthcare professionals at your medical center to train others to complete mandated case reviews | No Yes | No Yes |
| 1. Use educational institutions to train relevant healthcare professionals to complete mandated case reviews | No Yes | No Yes |

**Support Relevant Healthcare Professionals**

| **In the last 6-8 months, did your VA medical center employ any of these activities to support relevant healthcare professionals to promote completion of mandated case reviews of patients at very high risk for opioid-related adverse events?** | **Was this strategy used in the last 6-8 months?** | **[If yes] Was the above strategy implemented in direct response to *VHA Notice 2018-08*?** |
| --- | --- | --- |
| 1. Create new teams to conduct mandated case reviews of very high risk patients, for example interdisciplinary clinical workgroups | No Yes | No Yes |
| 1. Develop resource-sharing agreements with organizations or groups that have resources to help implement mandated case reviews | No Yes | No Yes |
| 1. Provide ongoing data to relevant healthcare professionals to facilitate the completion of mandated case reviews | No Yes | No Yes |
| 1. Develop reminders for relevant healthcare professionals to complete mandated case reviews of very high risk patients. Reminders could be delivered verbally, on paper, or electronically | No Yes | No Yes |
| 1. Revise professional roles to facilitate completion of mandated case reviews, including removing barriers or expanding roles, for example allow pharmacists to conduct the reviews | No Yes | No Yes |

**Engage Consumers**

| **In the last 6-8 months, did your VA medical center use any of these strategies to engage consumers (e.g., patients who are prescribed opioids and/or their family/caregivers) to promote completion of mandated case reviews of patients at very high risk for opioid-related adverse events?** | **Was this strategy used in the last 6-8 months?** | **[If yes] Was the above strategy implemented in direct response to *VHA Notice 2018-08?*** |
| --- | --- | --- |
| 1. Encourage patients to prompt relevant healthcare professionals to complete mandated case reviews | No Yes | No Yes |
| 1. Intervene with patients or family members to promote acceptance of having their case reviewed | No Yes | No Yes |
| 1. Involve patients or family members in the effort to ensure that mandated case reviews are completed | No Yes | No Yes |
| 1. Engage in efforts to prepare patients to be active participants in getting their cases reviewed | No Yes | No Yes |
| 1. Use local announcements, newsletters, or other media strategies to reach large numbers of patients or relevant healthcare professionals about completing mandated case reviews of very high risk patients | No Yes | No Yes |

**Utilize Financial Strategies**

| **In the last 6-8 months, did your VA medical center use any of these financial strategies to promote completion of mandated case reviews of patients at very high risk for opioid-related adverse events?** | **Was this strategy used in the last 6-8 months?** | **[If yes] Was the above strategy implemented in direct response to *VHA Notice 2018-08*?** |
| --- | --- | --- |
| 1. Acquire new or use existing funding to facilitate the completion of mandated case reviews of very high risk patients | No Yes | No Yes |
| 1. Use financial incentives to promote completion of mandated case reviews | No Yes | No Yes |
| 1. Add new financial penalties for failure to complete mandated case reviews, for example reduce pay or deny standard bonuses | No Yes | No Yes |
| 1. Pursue new funding mechanisms, like grants or contracts, to support the completion of mandated case reviews | No Yes | No Yes |
| 1. Make it easier to account for and document workload involved in completing mandated case reviews | No Yes | No Yes |
| 1. Use payment-related approaches other than those already mentioned to motivate completion of mandated case reviews | No Yes | No Yes |

**Change Infrastructure**

| **In the last 6-8 months, did your VA medical center use any of these infrastructure changes to promote completion of mandated case reviews of patients at very high risk for opioid-related adverse events?** | **Was this strategy used in the last 6-8 months?** | **[If yes] Was the above strategy implemented in direct response to *VHA Notice 2018-08*?** |
| --- | --- | --- |
| 1. Work with relevant accrediting bodies and professional membership organizations to encourage or require the use of case reviews of very high risk patients | No Yes | No Yes |
| 1. Change physical structure or availability of equipment to encourage completion of mandated case reviews | No Yes | No Yes |
| 1. Make change(s) to the standard STORM case-review note template in CPRS to make it easier to document the case reviews | No Yes | No Yes |
| 1. Change the location where mandated case reviews are completed, for example provide an interdisciplinary setting to complete case reviews | No Yes | No Yes |
| 1. Encourage an existing credentialing or licensing organization to certify relevant healthcare professionals in the completion of mandated case reviews or create a group with the authority to do so | No Yes | No Yes |
| 1. Have local leaders, like the facility director, department or service line leaders, state publicly that completing the mandated case reviews is a priority | No Yes | No Yes |

**Demographics**

Please answer the following demographic questions about yourself that will help us describe all people we survey. This information is confidential.

| 1. Sex assigned at birth | Male  Female  Prefer not to answer |
| --- | --- |
| 1. Current gender identity (How do you describe yourself?) | Male  Female  Transgender  Do not identify as female, male, or transgender  Prefer not to answer |
| 1. Current age | 18-25 years  26-30 years  31-35 years  36-40 years  41-45 years  46-50 years  51-55 years  56-60 years  61-65 years  66-70 years  71-75 years  76 years or older  Prefer not to answer |
| 1. Ethnicity | Hispanic or Latino (Including Cuban, Mexican, Puerto Rican, South or Central American, or other Spanish culture or origin)  Not Hispanic or Latino  Prefer not to answer |
| 1. Race (select one or more) | American Indian or Alaskan Native (Origins in any of the original peoples of North, Central, or South America)  Asian (Origins in any of the original peoples of the Far East, Southeast Asia, or the Indian subcontinent)  Black or African American (Origins in any of the black racial groups of Africa)  Native Hawaiian or other Pacific Islander (Origins in any of the original peoples of Hawaii, Guam, Samoa, or other Pacific Islands)  White (Origins in any of the original peoples of Europe, the Middle East, or North Africa)  Other (please specify) _____________  Prefer not to answer |
| 1. Highest degree awarded | High School Diploma or GED  Associate’s Degree  Bachelor’s Degree  Master’s Degree  MD or DO  DNP  PhD  Multiple doctorate level degrees (please specify)  _______________  Other (please specify) __________  Prefer not to answer |
| 1. What is your VA job title? | ______________________________ |
| 1. What is your primary role in the VA? | Clinician  Pharmacist  Case Manager  Administrator  Researcher  Social Worker  Other (please specify) ____________________  Prefer not to answer |
| 1. How many years have you been in your primary role? | Less than 1 year  1-5 years  6-10 years  11-15 years  16-20 years  21-25 years  26-30 years  31 or more years  Prefer not to answer |
| 1. What other roles do you have in the VA? | Clinician  Pharmacist  Case Manager  Administrator  Researcher  Social Worker  Other (please specify) ____________________  Prefer not to answer  Not applicable |
| 1. How many years have you been with the VA overall? | Less than 1 year  1-5 years  6-10 years  11-15 years  16-20 years  21-25 years  26-30 years  31 or more years  Prefer not to answer |
| 1. How many hours per week do you see patients? | I do not see patients  Less than 5 hours  6-10 hours  11-15 hours  16-20 hours  21-25 hours  26-30 hours  31-35 hours  36-40 hours  Prefer not to answer |
| 1. Do you have opioid prescribing privileges? | Yes  No  Prefer not to answer |

Thank you for taking the time to complete this survey. If you have additional comments you find important to share, please enter them below.
